# Correction: Development and evaluation of rapid data-enabled access to routine clinical information to enhance early recruitment to the national clinical platform trial of COVID-19 community treatments

**DOI:** 10.1186/s13063-023-07606-4

**Published:** 2023-09-19

**Authors:** Caroline Cake, Emma Ogburn, Heather Pinches, Garry Coleman, David Seymour, Fran Woodard, Sinduja Manohar, Marjia Monsur, Martin Landray, Gaynor Dalton, Andrew D. Morris, Patrick F. Chinnery, UK COVID-19 National Core Studies Consortium, F. D. Richard Hobbs, Christopher Butler

**Affiliations:** 1 Health Data Research UK, Wellcome Trust, Gibbs Building, 215 Euston Road, London, NW1 2BE UK; 2 Nuffield Department of Primary Care Health Sciences, University of Oxford, Gibson Building 1St Floor, Radcliffe Observatory Quarter, Woodstock Road, Oxford, OX2 6GG UK; 3 NHS DigiTrials, Skipton House, 80 London Rd, Elephant and Castle, London, UK; 4 NHS Digital, Skipton House, 80 London Rd, Elephant and Castle, London, SE1 6LH UK; 5 DHSC, Department for Health and Social Care, 39 Victoria St, Westminster, London, SW1H 0EU UK; 6 HDR UK Oxford, Big Data Institute, Nuffield Department of Population Health, University of Oxford, Oxford, OX3 7LF UK; 7 Department of Clinical Neuroscience & Medical Research Council Mitochondrial Biology Unit, School of Clinical Medicine, University of Cambridge, Cambridge, CB2 0QQ UK

**Correction: BMC Trials 23, 62 (2022)**

**https://doi.org/10.1186/s13063-021-05965-4**

Following publication of the original article [1], we have been informed that a supplementary file was missing. This is now referenced at the end of the Results section (“Select rankings of factors, motivations, and barriers influencing research participation and retention varied by site (Additional File 2)”.

The original article has been corrected.
